# Metabolomic Analysis Revealed the Differences in Metabolites Between Three Different Sugarcane Stems and Leaves

**DOI:** 10.3390/metabo15050327

**Published:** 2025-05-15

**Authors:** Hongbo Lou, Linyan Xie, Xianhong Wang, Xianli Li, Lilian He, Fusheng Li

**Affiliations:** 1 College of Agronomy and Biotechnology, Yunnan Agricultural University, Kunming 650201, China; hongbo_lou123@163.com (H.L.); xly1977151909@163.com (L.X.); x.h_wang@163.com (X.W.); 18387272674@163.com (X.L.); 2 Sugarcane Research Institute, Yunnan Agricultural University, Kunming 650201, China; 3 College of Biological Sciences and Agronomy, Honghe University, Mengzi 661100, China

**Keywords:** sugarcane, metabolomics, differential metabolites, flavonoids, phenolic acids

## Abstract

**Background:** Sugarcane is an important sugar crop. Sugarcane stems are mainly used for sugar extraction, while leaves can only be burned as waste. However, sugarcane leaves can also produce a large number of secondary metabolites, and these metabolites have significant nutritional and pharmacological value. At present, there are few studies on sugarcane compounds. **Methods:** Therefore, the stems and leaves of three sugarcane varieties (Yacheng 89-159, Dianzhe 01-58, ROC22) were selected as experimental materials, and the compounds of stems and leaves of different sugarcane were studied using high-performance liquid chromatography. **Results:** Metabolomics analysis detected 1197 metabolites that could be broadly divided into 11 categories. Orthogonal partial least squares discriminant analysis identified metabolites that were differentially abundant across groups (stems and leaves within and across the three varieties). Flavonoids, phenolic acids, and lipids were the main differential metabolites. Notably, tricin-4′-O-(guaiacylglycerol)ether-7-O-glucoside, quercetin-3,4-O-di-glucoside, cyanidin-3-O-(6′′-O-malony)glucoside were significantly higher in the stems than in the leaves across all three varieties. The content of methylenesuccinic acid was higher in the leaves of Dianzhe 01-58 and ROC22. In the comparative analysis of the top 20 differential metabolites among different varieties, it was found that the metabolite content of stems and leaves of Yacheng 89-9 and ROC22 was significantly higher than that of Dianzhe 01-58. Next, KEGG analysis showed that these differential metabolites were mainly enriched in pathways related to flavonoid, phenylpropanoid, and isoflavonoid biosynthesis, as well as starch and sucrose metabolism. Leaves also had significantly fewer metabolites involved in starch and sucrose metabolism than stems did. **Conclusion:** In conclusion, this study provides a scientific basis for utilization of sugarcane compounds, laying a theoretical foundation for further processing of sugarcane by-products into higher-value materials.

## 1. Introduction

Sugarcane (*Saccharum officinarum* L.) is a solid herbaceous perennial and highly light-efficient C4 plant. Globally, it is the most important sugar and bioenergy crop [1]. China is a major player in sugarcane agriculture and sugar processing, with >85% of the planting area for sugar crops allocated to sugarcane, which accounts for >92% of total sugar [2]. In 2022, the area under sugarcane cultivation in China was 1.289 million hm^2^, producing 103.381 million tons of sugarcane (National Bureau of Statistics).

Waste products from sugarcane processing include bagasse, molasses, and sugarcane leaves; the latter comprises 20% of total crop mass [3]. Sugarcane leaves and bagasse are typically incinerated, causing considerable environmental pollution and spurring increased focus on waste recycling. Fortunately, sugarcane by-products are renewable resources, with relatively centralized origin and uniform harvesting time; these characteristics indicate that by-products can be readily developed and used if they have sufficient practical value [4]. Besides sugar, sugarcane has also been used to produce alcohol, paper, industrial raw materials, and fiber in livestock feed [5].

One intriguing direction of research in terms of using sugarcane by-products is as a form of treatment. In the Dictionary of Traditional Chinese Medicine, sugarcane has been commonly used to treat bladder stones, kidney stones, and gonorrhea [6]. In India, sugarcane juice is used to treat jaundice, bleeding, dysuria, anuria, and other urinary diseases [7]. Furthermore, native and traditional healers worldwide have recommended sugarcane juice for its diuretic properties [8,9]. A better knowledge of the active compounds within sugarcane is needed to verify and explain these effects. Besides being high in sugars (including polysaccharides), sugarcane is high in phenolic acids, flavonoids, phytosterols, triterpenes, anthocyanins, and other phytochemicals [5,10]. An analysis of leaves from different sugarcane genotypes identified 144 metabolites; 56 of these were phenolics (19) and flavones (25), with a predominance of isomeric flavone C-glycosides and some other compounds [11]. These metabolites likely explain the plant’s antibacterial, hypoglycemic, antitumor, and anti-neuroinflammatory activities, as well as its importance as a source of exogenous antioxidants in the human body [12,13,14]. Furthermore, the cosmetics industry has used sugarcane extracts to obtain phenolic compounds and other bioactive ingredients for their products [15]. However, few reports are available on the specific active constituents.

The widespread nature of sugarcane necessitates a comprehensive understanding of its endogenous compounds. Ultra-high-performance liquid chromatography tandem mass spectrometry (UPLC-MS/MS) is widely and effectively applied in differential analysis of metabolites, including in Chinese herbal medicines [16,17,18]. Hence, this study used UPLC-MS/MS to determine differential metabolite composition in the stems and leaves of three sugarcane cultivars (Yacheng 89-9, Dianzhe 01-58, and ROC22). Our results should clarify the active components of sugarcane, opening the path to their potential for medical use and in the cosmetics industry, fully utilizing sugarcane by-products to reduce environmental pollution.

## 2. Results

### 2.1. Analysis of Principal Components and Fractions of Metabolites in Different Sugarcane Varieties

**Figure 1:**
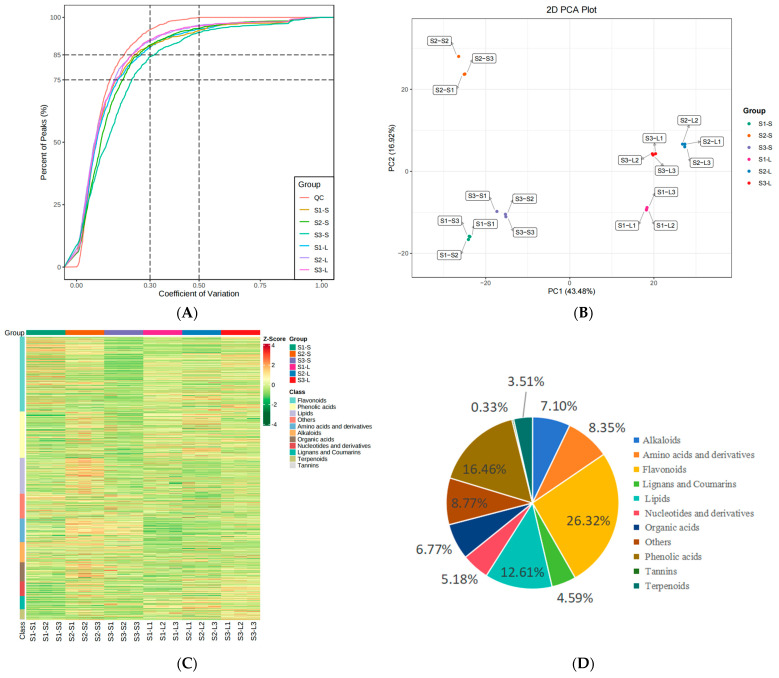
Qualitative and quantitative component analysis of metabolites in three sugarcane varieties. (**A**) Quality control (QC) sample with the coefficient of variation (CV) from LC-MS. (**B**) Principal component analysis. S1, Yacheng 89-9; S2, Dianzhe 01-58; S3, ROC22. S, stems; L, leaves. (**C**) Heatmap of identified metabolites. (**D**) Chemical classification of all identified metabolites.

The composition of common and differential metabolites in leaves and stems of three sugarcane varieties were determined with UPLC-ESI-MS/MS-based broad-targeted metabolomics (Figure 1A), with a quality control (QC) sample with Coefficient of Variation (CV) of LC-MC less than 0.3 accounting for more than 75% of the samples, indicating that the experimental data were very stable. Analysis of the total ion current (TIC) revealed that the TIC curve of detected metabolites had strong overlap. That is, retention time and peak intensity were consistent, indicating that the same metabolite had a stable signal across different time points (Figure S1). Next, PCA revealed between-group differences. First, leaf and stem metabolites were distinct from each other. Among the varieties, Yacheng 89-9 and ROC22 stems clustered together, distinct from Dianzhe 01-58 stems. However, Dianzhe 01-58 and ROC22 leaves clustered together, distinct from Yacheng 89-9 leaves. Respectively, PC1 and PC2 explained 43.48% and 16.92% of variance in metabolites (Figure 1B). The hierarchical cluster analysis (HCA) heatmap classified 18 samples into six groups (Figure 1C). Overall, we detected 1197 metabolites from the 18 sugarcane samples and categorized them into 11 groups; the most abundant groups were flavonoids (26.32%), phenolic acids (16.46%), lipids (12.61%), amino acids and derivatives (8.35%), and alkaloids (7.10%) (Figure 1D; Supplementary Data 1).

### 2.2. Orthogonal Partial Least Squares Discriminant Analysis

The predictive parameters of the orthogonal partial least squares discriminant analysis (OPLS-DA) model are R2X, R2Y, and Q2, where R2X and R2Y denote the model’s explanatory rate (goodness of fit) on the X and Y matrices, respectively, and Q2 denotes predictive ability. Model stability and reliability are higher when the three indices are closer to 1; Q2 > 0.5 represents a good model, while Q2 > 0.9 represents an excellent one.

We observed high predictability (Q2) and powerful goodness of fit (R2X, R2Y) between S1-S and S2-S (Q2 = 0.9987, R2X = 0.711, R2Y = 0.999; Figure S2A), S1-S and S3-S (Q2 = 0.98, R2X = 0.669, R2Y = 0.999; Figure S2B), S2-S and S3-S (Q2 = 0.983, R2X = 0.704, R2Y = 998; Figure S2C), S1-L and S2-L (Q2 = 0.984, R2X = 0.69, R2Y = 1; Figure S2D), S1-L and S3-L (Q2 = 0.984, R2X = 0.69, R2Y = 1; Figure S2E), S2-L and S3-L (Q2 = 0.982, R2X = 0.676, R2Y = 1; Figure S2F), S1-S and S1-L (Q2 = 0.993, R2X = 0.783, R2Y = 1; Figure S2G), S2-S and S2-L (Q2 = 0.966, R2X = 0.815, R2Y = 1; Figure S2H), as well as S3-S and S3-L (Q2 = 0.991, R2X = 0.759, R2Y = 1; Figure S2I). Hence, our model was both stable and reliable for identifying differential and abundant metabolites. Notably, the stems and leaves of all three varieties (S1-S, S2-S, S3-S, S1-L, S2-L, and S3-L) possessed distinct metabolic profiles.

### 2.3. Differential Metabolite Analysis

**Figure 2:**
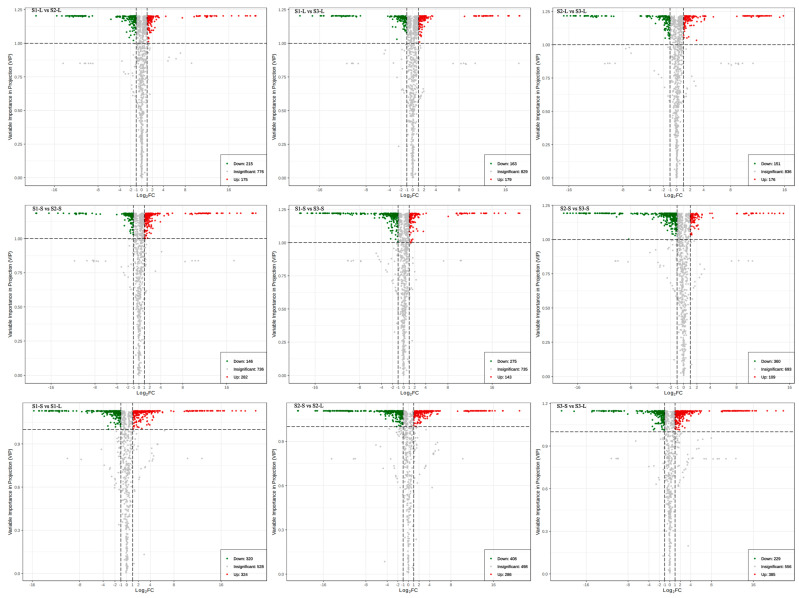
Differential metabolites among the six samples (stems and leaves of three sugarcane varieties): S1-S, S2-S, S3-S, S1-L, S2-L, and S3-L.

Based on OPLS-DA results, we selected metabolites with VIP > 1, FC ≥ 2, and FC ≤ 0.5 for further analysis. In leaves, screening yielded 390 differential metabolites (175 upregulated and 215 downregulated) between S1 and S2, mainly flavonoids (127), phenolic acids (70), lipids (66), alkaloids (27), and organic acids (23) (Figure 2; Table S1). Additionally, 342 differential metabolites (179 upregulated and 163 downregulated) were present between S1-L and S3-L, mainly flavonoids (99), phenolic acids (60), lipids (41), alkaloids (28), and amino acids and derivatives (24). Finally, 327 metabolites were differentially expressed (176 upregulated and 151 downregulated) between S2-L and S3-L, mainly flavonoids (92), phenolic acids (81), lipids (32), organic acids (21), and lignans and coumarin (21).

Next, we identified 428 stem metabolites that were differentially expressed between S1 and S2 (282 upregulated, 146 downregulated), comprising mainly flavonoids (122), phenolic acids (77), lipids (53), organic acids (33), and amino acids and derivatives (30). Between S1-S and S3-S, we found 418 differentially expressed metabolites (143 upregulated, 275 downregulated). These differential metabolites were mainly flavonoids (163), phenolic acids (78), lipids (58), lignans and coumarin (21). We also identified 469 differentially expressed metabolites (109 upregulated and 360 downregulated) between S2-S and S3-S. These largely comprised flavonoids (164), phenolic acids (88), lipids (70), alkaloids (27), and nucleotides and derivatives (22).

Between sugarcane leaves and stems, we found 644 differential metabolites (324 upregulated and 320 downregulated) across S1-S and S1-L, largely flavonoids (206), phenolic acids (115), lipids (61), alkaloids (49), and amino acids and derivatives (41). In S2, 694 metabolites were differentially expressed (286 upregulated, 408 downregulated) between stems and leaves, mainly flavonoids (209), phenolic acids (115), lipids (88), organic acids (49), and alkaloids (47). Finally, S3-S and S3-L differentially expressed 614 metabolites (385 upregulated, 229 downregulated), primarily flavonoids (200), phenolic acids (101), lipids (52), alkaloids (47), and organic acids (38).

The Wayne diagram analysis reveals the number of metabolites per group and their overlapping relationships across groups. The six samples varied in number of total metabolites (Figure S3), with the number of unique components in S1-L vs. S2-L, S1-L vs. S3-L, and S2-L vs. S3-L being 68, 47, and 59, respectively. The number of unique components in S1-S vs. S2-S, S1-S vs. S3-S, and S2-S vs. S3-S was 78, 45, and 54. Finally, the number of unique components in S1-S vs. S1-L, S2-S vs. S2-L, and S3-S vs. S3-L was 68, 127, and 84.

### 2.4. Analysis of Top 20 Metabolites After Multiple Comparisons Across Groups

**Figure 3:**
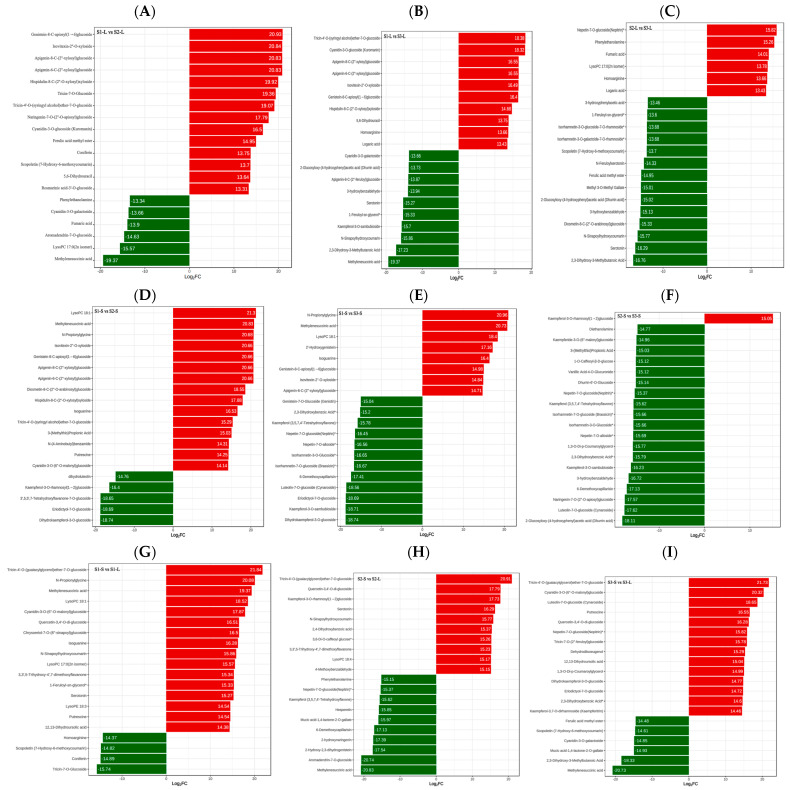
Analysis of the top 20 differentially expressed metabolites after multiple pairwise comparisons between groups. (**A**) S1-L vs. S2-L. (**B**) S1-L vs. S3-L. (**C**) S2-L vs. S3-L. (**D**) S1-S vs. S2-S. (**E**) S1-S vs. S3-S. (**F**) S2-S vs. S3-S. (**G**) S1-S vs. S1-L. (**H**) S2-S vs. S2-L. (**I**) S3-S vs. S3-L.

We performed multiple comparisons to identify the top 20 metabolites per pair of variety and plant part. First, we investigated leaf metabolites across the three varieties. Genistein-8-C-apiosyl(1→6)glucoside, isovitexin-2′′-O-xyloside, apigenin-8-C-(2′′-xylosyl)glucoside, apigenin-6-C-(2′′-xylosyl)glucoside, and hispidulin-8-C-(2′′-O-xylosyl)xyloside were significantly higher in S1-L than in S2-L. Cyanidin-3-O-galactoside, fumaric acid, aromadendrin-7-O-glucoside, lysoPC 17:0 (2n isomer), and methylenesuccinic acid were significantly higher in S2-L than in S1-L (Figure 3A). Tricin-4′-O-(syringyl alcohol)ether-7-O-glucoside, cyanidin-3-O-glucoside (Kuromanin), apigenin-8-C-(2′′-xylosyl)glucoside, apigenin-6-C-(2′′-xylosyl)glucoside, and isovitexin-2′′-O-xyloside were significantly higher in S1-L than in S3-L. 1-Feruloyl-sn-glycerol*, kaempferol-3-O-sambubioside, N-sinapoylhydroxycoumarin, 2,3-dihydroxy-3-methylbutanoic acid, and methylenesuccinic acid were significantly higher in S3-L than in S1-L (Figure 3B). Nepetin-7-O-glucoside (nepitrin)*, phenylethanolamine, fumaric acid, lysoPC 17:0 (2n isomer), and homoarginine were higher in S2-L than S3-L, whereas 3-hydroxybenzaldehyde, diosmetin-8-C-(2′′-O-arabinosyl)glucoside, N-sinapoylhydroxycoumarin, serotonin, and 2,3-dihydroxy-3-methylbutanoic acid were lower in S2-L (Figure 3C).

Next, we compared stem metabolites across the three varieties. LysoPC 18:1, methylenesuccinic acid, N-propionylglycine, isovitexin-2′′-O-xyloside, and genistein-8-C-apiosyl(1→6)glucoside were significantly higher in S1-S than in S2-S, whereas the reverse was true for dihydroluteolin, kaempferol-3-O-rhamnosyl(1→2)glucoside, 3′,5,5′,7-tetrahydroxyflavanone-7-O-glucoside, eriodictyol-7-O-glucoside, and dihydrokaempferol-3-O-glucoside (Figure 3D). When comparing S1-S and S3-S, N-propionylglycine, methylenesuccinic acid, lysoPC 18:1, 2′-hydroxygenistein, and isoguanine were significantly higher in S1-S, whereas 6-demethoxycapillarisin, luteolin-7-O-glucoside (cynaroside), eriodictyol-7-O-glucoside, kaempferol-3-O-sambubioside, and dihydrokaempferol-3-O-glucoside were significantly higher in S3-S (Figure 3E). Finally, comparisons of metabolites between S2-S and S3-S revealed that kaempferol-3-O-rhamnosyl(1→2) glucoside was significantly higher in S2-S, whereas 3-hydroxybenzaldehyde, 6-demethoxycapillarisin, naringenin-7-O-(2′′-O-apiosyl) glucoside, luteolin-7-O-glucoside (cynaroside), and 2-glucosyloxy-(4-hydroxyphenyl)acetic acid (dhurrin acid) were significantly higher in S3-S (Figure 3F).

We then compared stem versus leaf compounds within the same sugarcane variety. For Yacheng 89-9, tricin-4′-O-(guaiacylglycerol)ether-7-O-glucoside, N-propionylglycine methylenesuccinic acid, lysoPC 18:1, and cyanidin-3-O-(6′′-O-malonyl)glucoside were significantly higher in stems, while homoarginine, scopoletin (7-hydroxy-6- methoxycoumarin), coniferin, and tricin-7-O-glucoside were higher in leaves (Figure 3G). For Dianzhe 01-58, tricin-4′-O-(guaiacylglycerol)ether-7-O-glucoside, quercetin-3,4′-O-di-glucoside, kaempferol -3-O-rhamnosyl(1→2)glucoside, serotonin, and N-sinapoylhydroxycoumarin were significantly higher in stems, while 6-demethoxycapillarisin, 2-hydroxynaringenin, 2-hydroxy-2,3-dihydrogenistein, aromadendrin-7-O-glucoside, and methylenesuccinic acid were higher in leaves (Figure 3H). For ROC22, tricin-4′-O-(guaiacylglycerol)ether-7-O-glucoside, Cyanidin-3-O-(6′′-O-malonyl)glucoside, luteolin-7-O-glucoside (cynaroside), putrescine, and quercetin-3,4′-O-di-glucoside were higher in stems, whereas scopoletin (7-hydroxy-6-methoxycoumarin), cyanidin-3-O-galactoside, mucic acid-1,4-lactone-2-O-gallate, 2,3-dihydroxy-3-methylbutanoic acid, and methylenesuccinic acid were higher in leaves (Figure 3I).

### 2.5. KEGG Annotation and Enrichment Analysis

**Figure 4:**
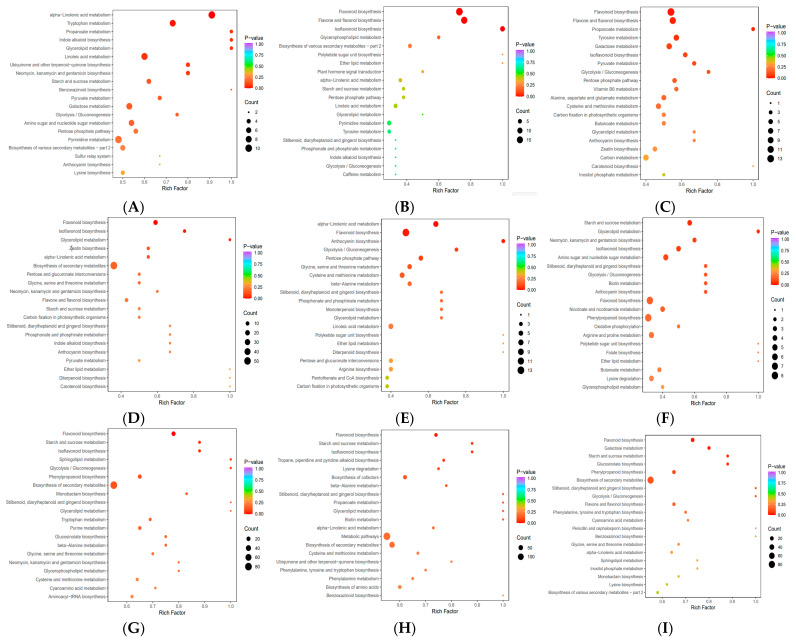
KEGG pathway enrichment analysis of differential metabolites across plant parts. (**A**) S1-S and S2-S. (**B**) S1-S and S3-S. (**C**) S2-S and S3-S. (**D**) S1-L and S2-L. (**E**) S1-L and S3-L. (**F**) S2-L and S3-L. (**G**) S1-S and S1-L. (**H**) S2-S and S2-L. (**I**) S3-S and S3-L.

We identified the top 20 enriched pathways after KEGG analysis and found that they were mostly related to six groups of metabolites (Figure 4A–I). First, we investigated stem metabolites that were differentially expressed across the three varieties. Between S1 and S2, differential metabolites were mainly enriched in alpha-linolenic acid, tryptophan, and linoleic acid metabolism (Figure 4A). Between S1 and S3, they were predominantly enriched in flavone, flavonoid, flavonol, and isoflavonoid biosynthesis (Figure 4B). Lastly, between S2 and S3, they were enriched in flavonoid, flavone, flavonol, and tyrosine biosynthesis, as well as galactose metabolism (Figure 4C).

Next, differentially expressed leaves metabolites across S1 and S2 were involved in secondary metabolite, flavonoid, and isoflavonoid biosynthesis (Figure 4D). Between S1 and S3, they were predominantly related to flavonoid biosynthesis, along with cysteine, methionine, and alpha-linolenic acid metabolism (Figure 4E). Between S2 and S3, they were enriched in flavonoid and phenylpropanoid synthesis, as well as amino sugar and nucleotide sugar metabolism (Figure 4F).

We then compared across plant parts within varieties. Differentially expressed metabolites between stems versus leaves in S1 were enriched in secondary metabolite, flavonoid, phenylpropanoid, and isoflavonoid biosynthesis, as well as starch and sucrose metabolism (Figure 4G). Differential metabolites in S2 were enriched in secondary metabolite, amino acid, cofactor, and flavonoid biosynthesis (Figure 4H). Differential metabolites in S3 were enriched in secondary metabolite, flavonoid phenylpropanoid, flavone, and flavonol biosynthesis, as well as galactose metabolism (Figure 4I).

To summarize, differential leaves metabolites across the three sugarcane varieties were mainly enriched in amino acid and aminoacyl-tRNA biosynthesis, along with linoleic acid, 2-oxocarboxylic acid, cysteine, and methionine metabolism. Differential stem metabolites were mainly enriched in biosynthesis of secondary metabolites, flavonoid biosynthesis, flavone and flavonol biosynthesis, aminoacyl—tRNA biosynthesis, and isoflavonoid biosynthesis.

### 2.6. Heat Map Analysis of Metabolites in Major Metabolic Pathways

**Figure 5:**
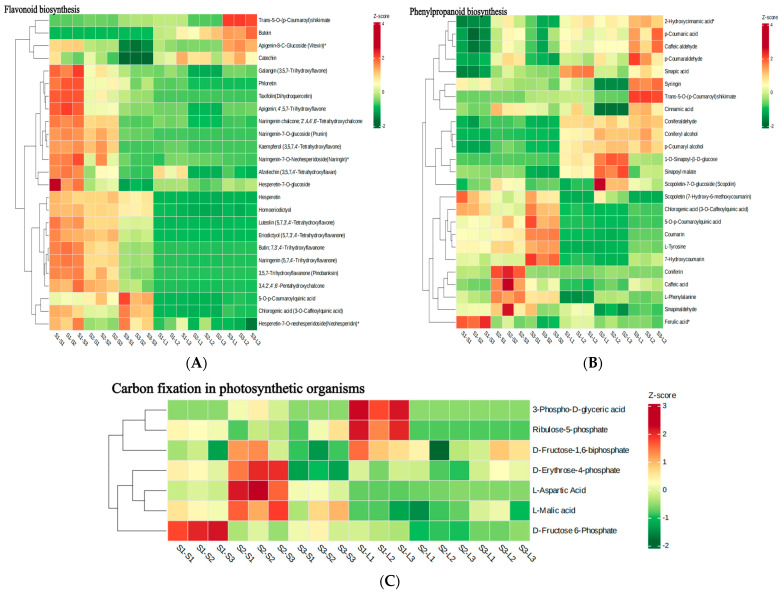

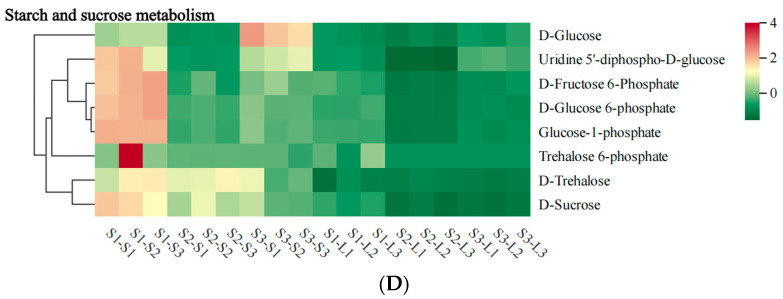
Heatmap of metabolites involved in major metabolic pathways across varieties and plant parts. (**A**) Flavonoid biosynthesis. (**B**) Phenylpropanoid biosynthesis. (**C**) Carbon fixation in photosynthetic organisms. (**D**) Starch and sucrose metabolism.

Based on the results of the KEGG analysis, we further analyzed metabolites related to flavonoid biosynthesis, phenylpropanoid biosynthesis, carbon fixation, and starch and sucrose metabolism. Flavonoid biosynthesis metabolites were more abundant in stems than in leaves, with Yacheng 89-9 stems in particular having significantly more of these metabolites than other groups (Figure 5A). In contrast, trans-5-O-(p-coumaroyl)shikimate, butein, apigenin-8-C-glucoside (vitexin), and catechin were higher in leaves. When we examined the phenylpropanoid biosynthesis pathway specifically, we observed that stems had more scopoletin (7-hydroxy-6-methoxycoumarin), chlorogenic acid (3-O-caffeoylquinic acid), 5-O-p-coumaroylquinic acid, coumarin, L-tyrosine, and 7-hydroxycoumarin than leaves. Leaves had more 2-hydroxycinnamic acid*, p-coumaric acid, caffeic aldehyde, coniferaldehyde, coniferyl alcohol, and p-coumaryl alcohol than stems (Figure 5B). In terms of metabolites from the carbon fixation pathway, Yacheng 89-9 stems expressed the most D-fructose 6-phosphate, Dianzhe 01-58 stems expressed the most D-fructose-1,6-biphosphate, D-erythrose-4-phosphate, L-aspartic acid, and L-malic acid, while Yacheng 89-159 leaves expressed the most 3-phospho-D-glyceric acid and ribulose-5-phosphate (Figure 5C). Lastly, leaves had significantly lower concentrations of metabolites in starch and sucrose metabolism than did stems (Figure 5D).

## 3. Discussion

Although sugarcane is known to offer certain health advantages, the bioactive compounds that are responsible for these benefits have not been sufficiently investigated; the few known compounds are mainly sugars, flavonoids, and phenolic acids [19,20,21]. Offering further clarity and precision on sugarcane compounds will help spur the sustainable reuse of sugarcane by-products, particularly given the high demand for it as an ingredient in traditional Chinese medicine [22]. Here, we used UPLC-MS/MS to detect 1197 metabolites (spanning 11 classes) in stems and leaves of three sugarcane varieties. We verified that the primary metabolites in sugarcane are flavonoids and phenolic acids. Moreover, we successfully identified differential metabolites between plant parts and across varieties. Our results greatly enrich our understanding of sugarcane chemical compositions.

We identified 351 metabolites common across all three sugarcane varieties that were differentially expressed between stems and leaves. Among this group of largely flavonoids (119) and phenolic acids (61), quercetin, kaempferol, coumaric acid, luteolin, caffeic acid, and ferulic acid were present in both stems and leaves (Supplementary Data S1). Tricin and its derivatives were particularly abundant throughout the plant in all three varieties. However, luteolin and quercetin were higher in stems than in leaves, while coumaric acid exhibited the opposite pattern. Kaempferol was detected in both S1 stems and leaves and in S2 stems, but not in S2 leaves or in any part of S3.

These compounds have notable medicinal properties. First, tricin exerts anti-obesity, lipid-lowering, anti-tumor, anti-skin photoaging, and antioxidant effects [23,24,25,26]. Luteolin has been linked to anti-inflammatory [27], hypoglycemic [28], anti-tumor [29], antibacterial [30], antiviral [31], and immunomodulatory effects [32]. Quercetin exhibits anti-cancer [33], antioxidant [34], anti-fibrotic [35], and anti-inflammatory properties [36]. Kaempferol and its glycosylated derivatives have protective functions on the heart and nervous system, possessing antibacterial, anti-diabetes, antioxidant, anti-inflammatory, and anti-tumor properties [37,38]. Quercetin, luteolin, and matrine are prominent active substances in Qinggan Huayu Granules, a traditional Chinese medication sometimes used to manage liver cancer symptoms [39]. Another formula called Shenkang injection contains quercetin, kaempferol, and luteolin; these active substances help to reduce acid reflux and promote blood circulation. Shenkang injection is also used to treat symptoms of chronic renal failure [40].

Coumaric acid has antioxidant, anti-inflammatory, and immunomodulatory effects. Most notably, the compound can promote tumor cell death, inhibit cancer growth and metastasis, alleviate atherosclerosis, lower drug cardiotoxicity, ameliorate diabetes, and exert neuroprotective effects [41,42,43,44,45]. Caffeic acid is another common active ingredient found in many ingredients of traditional Chinese medicines, such as thistle, dandelion, and Gastrodia elata. Caffeic acid tablets can mitigate bleeding, leading to its clinical use for diseases such as leukopenia or thrombocytopenia [46]. Likewise, ferulic acid is present in Chuanxiong (Ligusticum sinense ‘Chuanxiong’), Angelica, and Cimicifuga, other common ingredients in traditional Chinese medicine. Sodium ferulate tablets have been used to treat atherosclerosis, angina pectoris, cerebral infarction, and diabetes [47].

In addition, studies have shown that phenolic acids in sugarcane extracts have great potential for development in the cosmetics industry. Gallic acid possesses antioxidant and antimicrobial activity and has the ability to inhibit elastase, collagenase, and tyrosinase, as well as the ability to promote collagen synthesis [48]; ferulic acid can act as an anti-ageing agent by both inhibiting ROS, which has an impact in UV protection, cellular damage and in the production of inflammatory mediators [49]; p-coumaric acid was reported to have significant anti-inflammatory potential to inhibit NO production as well as decreased IL-6, IL-1β and TNFα [50]; luteolin can act as a substrate analog inhibitor against melanogenesis [51].

The top 20 metabolites between each treatment were analyzed, and the results showed that tricin-4′-O-(guaiacylglycerol)ether-7-O-glucoside, quercetin-3,4-O-di-glucoside, cyanidin-3-O-(6′′-O-malony)glucoside were significantly higher in the stems than in the leaves across all three varieties (Figure 3). Tricin-4′-O-(guaiacylglycerol)ether-7-O-glucoside has the function of lowering the active ingredient of uric acid, which can reduce the synthesis of uric acid by inhibiting the activity of hepatic xanthine oxidase, regulate the renal uric acid transporter, promote the excretion of uric acid, and reduce the kidney damage caused by hyperuricemia [52]. Cyanidin-3-O-glucoside (C3G), a choloroside, C3G is widely available in many fruits, vegetables, flowers, grains, and other plant foods and has been shown to have potential benefits for a variety of human neurological disorders [53]. Quercetin and its glycosides are the most abundant natural flavonoids found in the human diet. Quercetin has a variety of physiological activities, including antidiabetic, hypoglycemic, antihypertensive, and anti-inflammatory [54]. Traditional Chinese medicine believes that sugarcane juice is effective in quenching thirst, harmonizing the middle and broadening the stomach, generating fluids and moisturizing dryness, diuretic and nourishing, probably because the cane stems contain a large amount of compounds such as apigenin 7-O-glucoside and quercetin-3,4′-O-diglucoside. Methylenesuccinic acid was higher in the leaves of Dianzhe 01-58 and ROC22, while it was higher in the stems of Yacheng 89-9. Methylenesuccinic acid has powerful immunomodulatory activity, playing an important role in regulating energy metabolism, anti-inflammatory, antioxidant and various immune responses, and also exerting biological effects on almost all organs, which has good prospects for application in medicine and animal husbandry [55]. In the comparative analysis of varieties, it was found that there were significant differences in the top 20 metabolites between different treatments. The metabolite contents of stems and leaves of Yacheng 89-9 and ROC22 were significantly higher than those of Dianzhe 01-58, which may be the difference between varieties. Further research is needed on their differences.

The results of KEGG analysis revealed that differential sugarcane metabolites were mainly enriched in flavonoid, phenylpropanoid, flavone, and flavonol biosynthesis. Phenylpropane metabolism and flavonoid biosynthesis pathways are mainly associated with plant stress tolerance. In particular, flavonoids, the dominant compound in sugarcane, have important roles in plant growth and development [56]. Therefore, we further investigated the phenylpropanoid metabolism and flavonoid biosynthesis pathways, revealing that related metabolites were more abundant in Yacheng 89-9 stems than in the other two varieties. The changes in the accumulation of metabolites related to phenylpropanoids and flavonoid biosynthesis pathways are potential molecular factors for the enhanced resistance and ratooning of sugarcane varieties [57]. Yacheng 89-9 is usually used as one of the parent materials in sugarcane breeding, and its hybridization with Saccharum rufipilum Steud. produces Dianzhe 01-58. Sugar accumulation in sugarcane is a complex regulated process, involving the photosynthesis-centered carbon assimilation network, the carbohydrate metabolism network (sugar synthesis and degradation), and the sugar transport network, all mediated by regulatory molecules [58]. Hence, altering gene expression and metabolite accumulation in pathways associated with carbon fixation and carbohydrate metabolism are key to increasing sugarcane yield and sucrose content [57]. Notably, we observed that Yacheng 89-9 stems expressed the highest amount of D-fructose 6-phosphate, a metabolite involved in both carbon fixation and carbohydrate metabolism. Additionally, we found that leaves had fewer metabolites related to starch and sucrose metabolism than stems did, likely explaining the higher sugar content in stems.

Sugarcane is an important sugar crop in China. A large amount of straw is produced in the process of sugarcane production. The fiber content of sugarcane straw is high, and it does not easily decay naturally in that year. The number of diseases, insects, and grass sources is easily increased in the residual field. The traditional treatment of sugarcane straw is simple open burning. Although this method is simple and efficient, the large amount of carbon dioxide gas and soot particles emitted to the atmosphere not only aggravates the global greenhouse effect, but also poses a hazard to human respiratory health. The availability of these secondary metabolites, such as flavonoids, phenolic acids, and organic acids, in sugarcane opens up many possibilities for their use in various industries, including pharmaceuticals, nutritional health products, cosmetics, and agriculture. The extraction and utilization of these valuable compounds from sugarcane not only provides economic benefits, but also contributes to the sustainable use of this multifunctional plant.

## 4. Materials and Methods

### 4.1. Plant Materials

The test materials were Yacheng 89-9 (S1), Dianzhe 01-58 (S2) and ROC22 (S3). These three sugarcane varieties were planted in the same land of the Sugarcane Research Institute of Yunnan Agricultural University (102°45′ E, 25°8′ N). The soil organic matter content was 29.81 g/kg, organic carbon content was 17.32 g/kg, total nitrogen content was 1.48 g/kg, cation exchange capacity was 3.1 mol/kg, total potassium content was 7.87 g/kg, available potassium content was 185.68 mg/kg, total phosphorus content was 1.45 g/kg, available phosphorus content was 2.21 mg/kg, pH value was 6.48. The field management levels of the three sugarcane varieties from sowing to sampling were the same. Sampling of stems (S) and leaves (L) was performed at the node stage. Plant samples were preserved in liquid nitrogen and then sent to Metware Biotechnology (Wuhan, China) for sequencing. Six groups (S1-S, S2-S, S3-S, S1-L, S2-L, and S3-L) were analyzed independently. Each analysis involved three biological replicates, providing a total of 18 samples.

### 4.2. Sample Preparation and Extraction

The collected sugarcane samples were removed from the refrigerator at −80 °C and then vacuum freeze-dried. After freeze-drying, 70% methanol-containing internal standard extract was added according to the ratio of 30 times of concentration (for example, 300 μL extractant was added to 9 mL sample after freeze-drying, and 200 μL extractant was added to 6 mL sample after freeze-drying). After vortexing for 15 min, sonicate for 10 min in an ice-water bath and centrifuge for 3 min at 4 °C at 12,000 r/min. The supernatant was filtered with 0.22 μm microporous membrane and stored in the injection bottle for LC–MS/MS detection.

### 4.3. UPLC Conditions

The sample extracts were analyzed using a system (UPLC, ExionLC™ AD, https://sciex.com.cn/, accessed on 30 July 2022) and a tandem mass spectrometry system (https://sciex.com.cn/, accessed on 30 July 2022). The UPLC conditions were as follows: the column as Agilent SB-C18 (1.8 μm, 2.1 mm × 100 mm), column temperature 40 °C, injection volume 2 μL, flow rate was 0.35 mL/min. The separation of sugarcane root secretion fractions was carried out using gradient elution with the following elution procedure: at 0.00 min the solution B was 5%, the solution B increased linearly to 95% within 9.00 min and was maintained at 95% for 1 min, and from 10.00 to 11.10 min, the solution B was reduced to 5% and equilibrated at 5% for up to 14 min. Comparison of mass spectrometry databases to determine composition [59].

### 4.4. Qualitative and Quantitative Metabolite Analyses

Based on the MWDB database, qualitative substance assessment was performed using secondary mass spectrometry. Metabolites from different samples were quantified using the MRM mode (see Section 2.3), then subjected to peak-area integration and integration correction [60].

### 4.5. Sample Quality Control Analysis

Extracts were mixed to generate quality control (QC) samples. A single QC sample was included after each set of 10 test samples to track consistency in metabolite extraction and detection. Specifically, overlapping display analysis was performed on total ion flow diagrams from mass spectrometry detection of QC samples [61,62].

### 4.6. Multivariate Statistical Analysis

A model was established with multivariate statistical analysis, using the integrated statistical prcomp effects in R (www.rproject.org/, accessed on 30 July 2022) for illustrating between-group differences [63,64]. Heat maps were drawn with pheatmap in R. Hierarchical clustering was also performed on metabolite accumulation across samples. Next, elements of both independent and dependent variables were isolated using orthogonal partial least squares discriminant analysis (OPLS-DA) for differential variable screening [61,65]. Based on OPLS-DA results, metabolites were screened based on their variable importance in projection (VIP) indices, along with p-values and fold change [65]. Differences between control and treatments were considered significant with fold change ≥2 or ≤0.5 and VIP ≥1. Differential metabolites were detected using Venn diagrams, along with additional calibration and annotation via the KEGG database (https://www.kegg.jp/kegg/compound/, accessed on 30 July 2022) [66].

## 5. Conclusions

This study used UPLC-MS/MS to identify stem and leaf metabolites across three sugarcane varieties. After detecting 1197 metabolites, we verified that the main secondary metabolites in sugarcane are flavonoids and phenolic acids. Stems and leaves, as well as the individual varieties, differed significantly in metabolite composition. Flavonoids, phenolic acids, and lipids were the main differential metabolites and putative bioactive compounds, most of them also showed higher relative concentrations in the diffrent parts among the three sugarcane varieties. In the comparative analysis of varieties, it was found that there were significant differences in the top 20 metabolites among different treatments. The contents of metabolites in the stems and leaves of Yacheng 89-9 and ROC22 were significantly higher than those of Dianzhe 01-58. When we performed KEGG pathway analysis on these differential metabolites, we found that they were significantly enriched pathways related to biosynthesis of secondary metabolites, flavonoids, phenylpropanoids, flavones, and flavonols. Then, we found that the content of metabolites in stems was more abundant than that in leaves in the flavonoid biosynthetic metabolite pathway; in particular, the content of these metabolites in stems of Yacheng 89-9 was significantly higher than that in other groups. Leaves had significantly lower concentrations of metabolites in starch and sucrose metabolism than did stems. The results of this study clarified the chemical composition and medicinal value of sugarcane stems and leaves, providing an empirical foundation for efficient processing of sugarcane by-products into higher-value goods.

## Data Availability

The original contributions presented in this study are included in the article. Further inquiries can be directed to the corresponding authors.

## Supplementary Materials

The following supporting information can be downloaded at: https://www.mdpi.com/article/10.3390/metabo15050327/s1, Figure S1. Multipeak mass spectral chromatogram of metabolites acquired in negative ion mode (A) and positive ion mode (B). Figure S2. OPLS-DA scores plots between the different comparison groups. The horizontal coordinate indicates the predicted principal component, with the horizontal direction showing the gap between groups; the vertical coordinate indicates the orthogonal principal component, with the vertical direction showing the gap within groups; and the percentage indicates the rate at which the component explains the dataset. (A) S1-L and S2-L. (B) S1-L and S3-L. (C) S2-L and S3-L. (D) S1-S and S1-L. (E) S2-S and S2-L. (F) S3-S and S3-L. (G) S1-S and S2-S. (H) S1-S and S3-S. (I) S2-S and S3-S. Figure S3. Venn diagrams of the differential metabolites in different comparison groups. A: Venn diagram of differential metabolites between leaves of three sugarcane varieties. B: Wayne diagram of differential metabolites between stems of three sugarcane varieties. C: Wayne diagram of differential metabolites between stems and leaves of the same sugarcane variety. Table S1. Classification and quantity of differential metabolites between different treatments

## Abbreviations

MRM, multi-reaction monitoring; OPLS-DA, orthogonal partial least squares discriminant analysis; PCA, principal component analysis; QC, quality control; UPLC-MS/MS, ultra-high-performance liquid chromatography tandem mass spectrometry; VIP, variable importance in projection.
