# Supplementary material for: Metabolomic Analysis Revealed the Differences in Metabolites Between Three Different Sugarcane Stems and Leaves
Source: Metabolites. 2025 May 15;15(5):327. doi: 10.3390/metabo15050327 (PMC12114123; doi:10.3390/metabo15050327)
Supplement: Supplementary file 1 [file metabolites-15-00327-s001.zip › Supplementary Material.pdf]

Supporting Information

Metabolomic Analysis Revealed the Differences in Metabolites Between Three Different Sugarcane Stems and Leaves

Hongbo Lou [1,2,†], Linyan Xie [1,3,†], Xianhong Wang [1,2], Xianli Li [1], Lilian He [1,2,*] and Fusheng Li [1,2,*]

[1] College of Agronomy and Biotechnology, Yunnan Agricultural University, Kunming 650201, China; hongbo_lou123@163.com (H.L.); xly1977151909@163.com (L.X.); x.h_wang@163.com (X.W.); 18387272674@163.com (X.L.)
[2] Sugarcane Research Institute, Yunnan Agricultural University, Kunming 650201, China
[3] College of Biological Sciences and Agronomy, Honghe University, Mengzi 661100, China
* Correspondence: 1990017@ynau.edu.cn (F.L.); 1993019@ynau.edu.cn (L.H.)
† These authors contributed equally to this work.

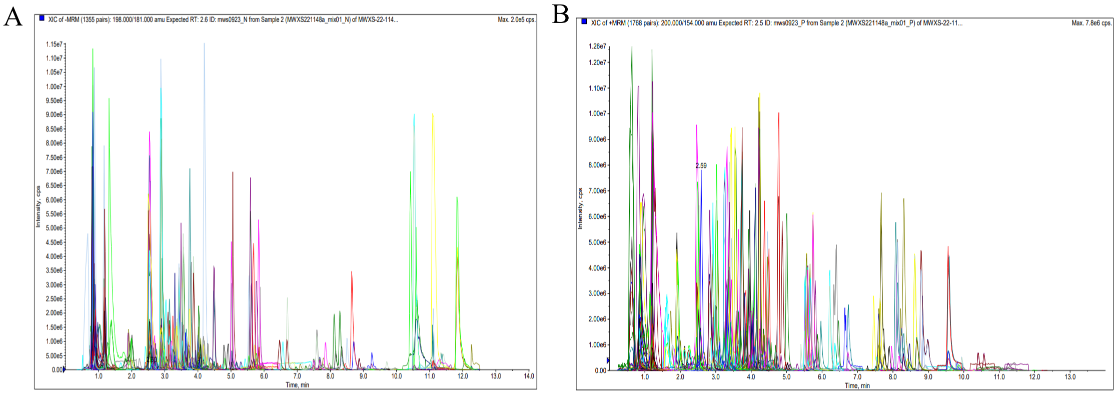

Figure. S1 Multipeak mass spectral chromatogram of metabolites acquired in negative ion mode (A) and positive ion mode (B).

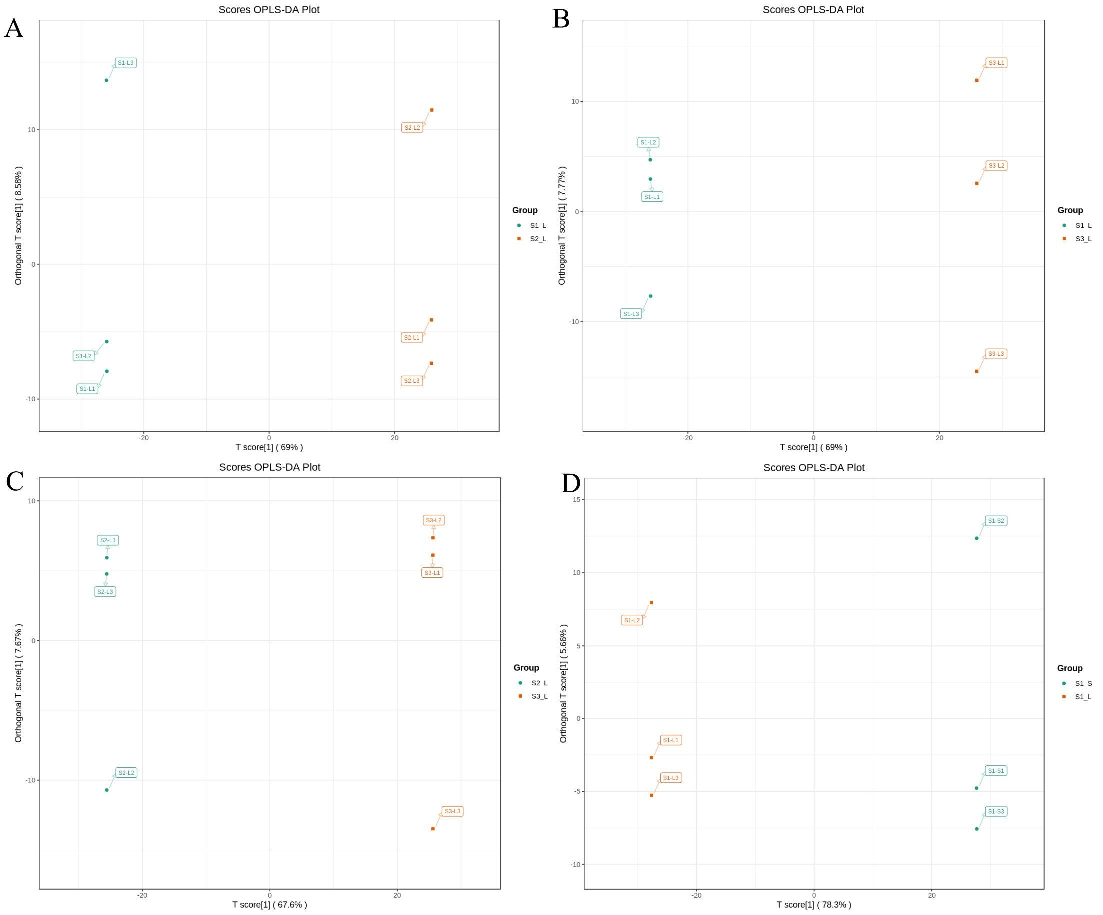

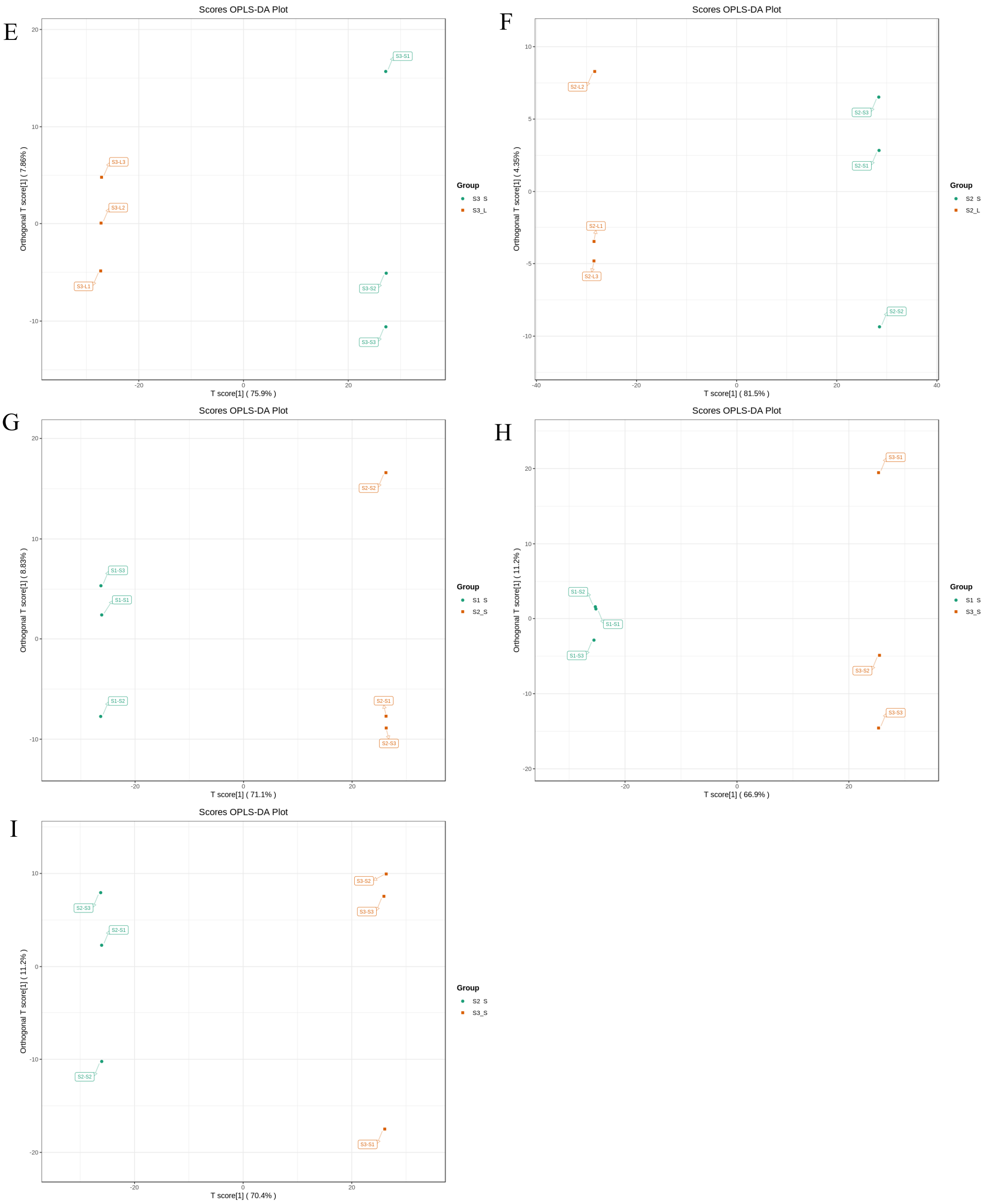

Figure. S2 OPLS-DA scores plots between the different comparison groups. The horizontal coordinate indicates the predicted principal component, with the horizontal direction showing the gap between groups; the vertical coordinate indicates the orthogonal principal component, with the vertical direction showing the gap within groups; and the percentage indicates the rate at which the component explains the dataset. (A) S1-L and S2-L, (B) S1-L and S3-L, (C) S2-L and S3-L, (D) S1-S and S1-L, (E) S2-S and S2-L, (F) S3-S and S3-L, (G) S1-S and S2-S, (H) S1-S and S3-S, (I) S2-S and S3-S.

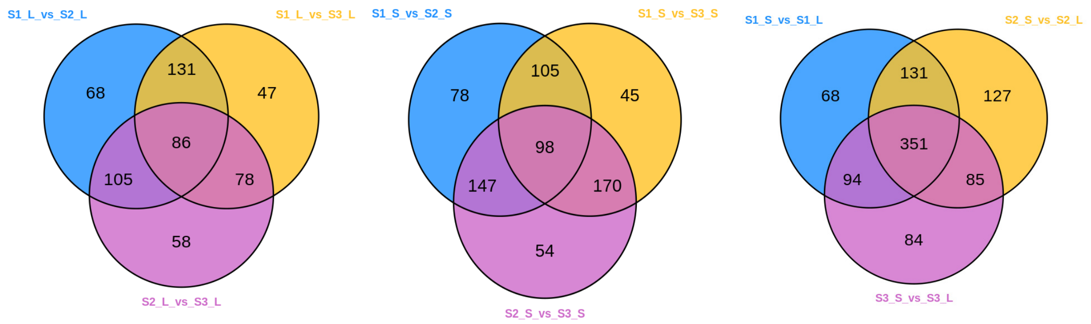

Figure. S3 Venn diagrams of the differential metabolites in different comparison groups.A: Venn diagram of differential metabolites between leaves of three sugarcane varieties. B: Wayne diagram of differential metabolites between stems of three sugarcane varieties. C: Wayne diagram of differential metabolites between stems and leaves of the same sugarcane variety.

Table S1 Classification and quantity of differential metabolites between different treatments

	Flavonoids	Phenolic acids	Organic acids	Alkaloids	Nucleotides and derivatives	Amino acids and derivatives	Lipids	Lignans and Coumarins	Terpenoids	Tannins	Others	total
S1_S vs S2_S	122	77	33	29	22	30	53	17	14	1	30	428
S1_S vs S3_S	163	78	13	19	16	19	58	21	7	1	23	418
S2_S vs S3_S	164	88	21	27	22	13	70	15	16	2	31	469
S1_L vs S2_L	127	70	23	27	22	16	66	13	10	0	16	390
S1_L vs S3_L	99	60	22	28	15	24	41	19	15	1	18	342
S2_L vs S3_L	92	81	21	20	12	15	32	21	14	1	18	327
S1_S vs S1_L	206	115	30	49	33	41	61	37	20	2	50	644
S2_S vs S2_L	209	115	49	47	26	42	88	35	27	3	53	694
S3_S vs S3_L	200	101	38	47	27	35	52	34	26	2	52	614
